# The complete chloroplast genome of *Digitaria sanguinalis* (Graminales: Gramineae)

**DOI:** 10.1080/23802359.2022.2087552

**Published:** 2022-06-23

**Authors:** You Liu, Chenzhong Jin, Xuejiao Zhang, Yunyun Zhou, Yihong Hu

**Affiliations:** Collaborative Innovation Center for Field Weeds Control, Hunan University of Humanities, Science and Technology, Loudi, Hunan, China

**Keywords:** Chloroplast genome, *Digitaria sanguinalis*, Gramineae, phylogenetic analysis

## Abstract

*Digitaria sanguinalis* (Linnaeus) Scopoli 1722 is an annual herbal plant that has important medicinal and ecological value. The chloroplast genome was 138,079 bp in length. In total, 129 genes were predicted, including 82 protein-coding genes, 39 tRNA genes, and 8 rRNA genes. The overall AT content of the genome was 61.39%. The phylogenetic analysis showed that *D. sanguinalis* and *D*. *glauca* formed a base clade in Panicoideae close to *Thyridolepis xerophila*. This study will help to understand the genetic diversity of the *Digitaria* plants.

*Digitaria sanguinalis* (Linnaeus) Scopoli 1722 is an annual herbal plant in Gramineae distributed in the temperate, subtropical, and tropical regions. Its seeds are edible, and its whole plant can be used as forage and traditional medicine (Ibrahim et al. 2019). Recent researches revealed that *D. sanguinalis* is a potential candidate for heavy metal excluder in ecological remediation (Wang et al. 2020). As an important wild plant source, *D. sanguinalis* taxon and functional traits have been attracted attention. However, limited information has been achieved on the chloroplast genome of this species till now. In this study, the *D. sanguinalis* chloroplast genome was sequenced and assembled. It would provide useful information for the phylogeny of *Digitaria* plants.

The material was collected from Louxing District, Loudi City (E111°58′47″, N27°40′45″), Hunan, China with permission of Loudi Agricultural Institute. The voucher specimen H02125 is stored in the herbarium of Hunan University of Humanities, Science and Technology (Xianzhi Ni, 2209833409@qq.com). Total DNA was extracted from the fresh leaves using CTAB method. The chloroplast genome was sequenced using Illumina NovaSeq 6000 and assembled using SPAdes3.10.1 with a *k*-mer gradient of 55, 87, and 121 with *Whiteochloa capillipes* (GenBank: KU291487) as reference (Bankevich et al. 2012). CDS, rRNA, and tRNA genes were predicted by Prodigal2.6.3, Hmmer3.1b2, and Aragorn1.2.38, respectively (Ju et al. 2020).

The complete chloroplast genome of *D. sanguinalis* was a circular structure of 138,079 bp in total length with 61.39% AT content. It had a large single-copy region of 80,131 bp and a small single-copy region of 12,502 bp, separated by a pair of inverted repeats of 22,723 bp each. Besides, 129 genes were annotated in total in the chloroplast genome, including 82 protein-coding genes, 39 tRNA genes, and 8 rRNA genes.

To reveal the *D. sanguinalis* phylogeny, a maximum-likelihood (ML) phylogenetic tree was constructed using 9 complete chloroplast genome sequences. The sequences except *D. sanguinalis* were downloaded from GenBank and aligned using ClustalW. The ML tree was produced by MEGA11.0.9 using 1,000 bootstrap replicates (Tamura et al. 2021). The phylogenetic tree showed that *D. sanguinalis* and *D*. *glauca* formed a base clade in Panicoideae which was close to *Thyridolepis xerophila* (Figure 1). Comparison with this genome to previously published data showed a high level of gene synteny with *D. glauca* (GenBank: MK593550). This study will help to understand the genetic diversity of the *Digitaria* plants.

**Figure 1.**
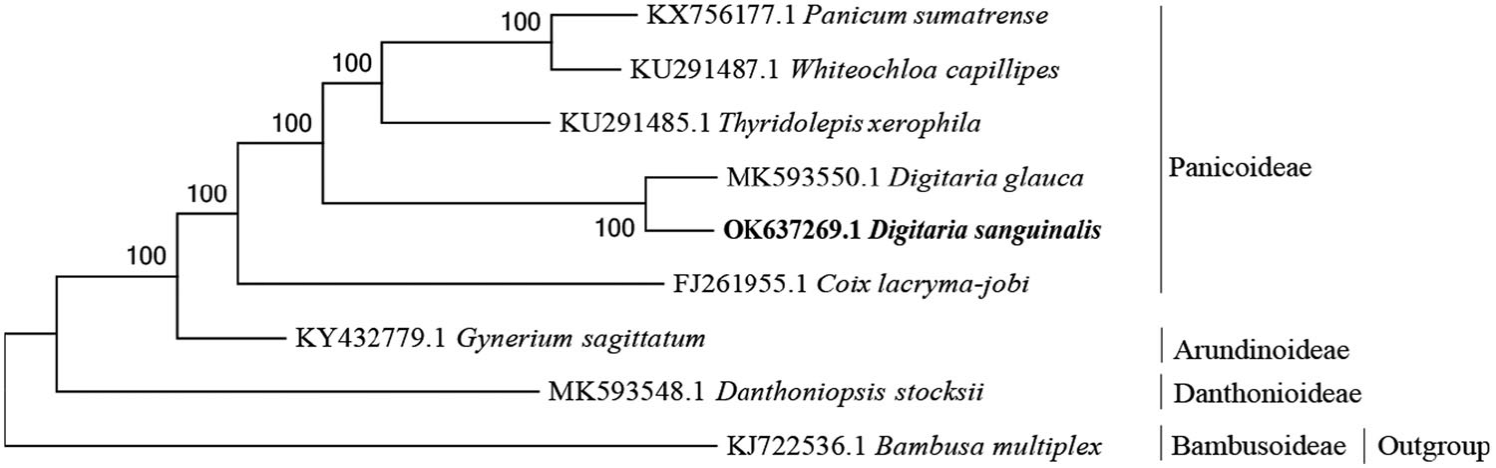
Maximum-likelihood phylogenetic tree based on 9 complete chloroplast genomes (bootstrap repeat is 1000).

## Author contributions

YL performed the main experiment, CJ collected samples, XZ analyzed the data, YZ validated the experiment, YH conceived the experiment, and all authors agree to be accountable for all aspects of the work.

## Data Availability

The genome sequence data that support the findings are openly available in GenBank of NCBI at https://www.ncbi.nlm.nih.gov/ under the accession number OK637269. The associated BioProject, SRA, and Biosample numbers are PRJNA774429, SRX12773576, and SAMN22563689, respectively.
